# Association between the ABO locus and hematological traits in Korean

**DOI:** 10.1186/1471-2156-13-78

**Published:** 2012-09-10

**Authors:** Kyung-Won Hong, Sanghoon Moon, Young Jin Kim, Yun Kyoung Kim, Dong-Joon Kim, Cheong-sik Kim, Sung Soo Kim, Bong-Jo Kim

**Affiliations:** 1 Center for Genome Science, Korea National Institute of Health, Osong, Chung-Buk, 363-951, South Korea

**Keywords:** ABO, GWAS, CNV, Hematological trait, Korean

## Abstract

**Background:**

Recently, genome-wide association studies identified a pleiotropic gene locus, *ABO*, as being significantly associated with hematological traits. To confirm the effects of *ABO* on hematological traits, we examined the link between the *ABO* locus and hematological traits in Korean population-based cohorts.

**Results:**

Six tagging SNPs for *ABO* were analyzed with regard to their effects on hematological traits [white blood cell count (WBC), red blood cell count (RBC), platelet (Plat), mean corpuscular volume (MCV), and mean corpuscular haemoglobin concentration (MCHC)]. Linear regression analyses were performed, controlling for recruitment center, sex, and age as covariates. Of the 6 tagging SNPs, 3 (rs2073823, rs8176720, and rs495828) and 3 (rs2073823, rs8176717, and rs687289) were significantly associated with RBC and MCV, respectively (Bonferroni correction p-value criteria < 0.05/6 = 0.008). rs2073823 and a reported SNP (rs8176746), as well as rs495828 and a reported SNP (rs651007), showed perfect linkage disequilibrium status (*r*^2^s = 0.99). Of the remaining 3 SNPs (rs8176720, rs8176717 and rs687289), rs8176717 generated an independent signal with moderate *p*-value (= 0.045) when it was adjusted for by rs2073823 (the most significant SNP). We also identified a copy number variation (CNV) that was tagged by the SNP rs8176717, the minor allele of which correlated with the deletion allele of CNV. Our haplotype analysis indicated that the haplotype that contained the CNV deletion was significantly associated with MCV (*β* ± se = 0.363 ± 0.118, *p* =2.09 × 10^-3^).

**Conclusions:**

Our findings confirm that *ABO* is one of the genetic factors that are associated with hematological traits in the Korean population. This result is notable, because GWASs fail to evaluate the link between a CNV and phenotype traits.

## Background

The ABO gene encodes isoforms for terminal glycosyltransferases, which transfer N-acetylgalactosamine and galactose to a common precursor (H substance), and lies on chromosome 9q34.2, containing 7 exons [1]. Exon 7 contains a domain that distinguishes between the A and B activities of the glycosyltransferase [2]. Several genomewide association studies (GWASs) have identified *ABO* as a candidate marker of the risk for coronary artery disease (CAD) [3], in addition to established CAD markers (sE-selectin, sP-selectin, and s-ICAM1) [4-6].

Hematological traits, such as red blood cell count (RBC), white blood cell count (WBC), platelet number (Plat), hemoglobin level (Hb), and hematocrit (Hct), are measured routinely to diagnose and monitor hematologic diseases and ascertain overall patient health. Recent GWASs on hematological traits have been reported for Caucasian [7], Japanese [8], and African-American [9] cohorts. These studies have identified more than 30 loci that carry common DNA polymorphisms that are linked to hematological traits.

The pleiotropic gene *ABO* correlated significantly with hematological traits in a Japanese [8] and African-American study [9], 3 SNPs of which (rs8176746, rs651007, rs495828) were reported in previous GWASs. rs8176746 is a nonsynonymous SNP and a deterministic variant of the B-type blood group [10]. rs651007 and rs495828 lie in the promoter region and are associated with CAD [4]. To confirm the effects of *ABO* on hematological traits, we examined the link between the *ABO* locus and hematological traits in Korean population-based cohorts.

## Results

### Hematological traits

The population characteristics and mean hematological traits are described in Table 1. Six hematological traits [WBC, RBC, Hb, Hct, Plat, and mean corpuscular volume (MCV)] were measured experimentally, and 2 other traits [mean corpuscular haemoglobin (MCH) and mean corpuscular hemoglobin concentration (MCHC)] were calculated using the RBC, Hb, and Hct values. Among hematological traits, RBC correlated with Hb and Hct, with Pearson’s *r* = 0.86 and 0.84, respectively. Also, MCV was linked to MCH, with Pearson’s *r* = 0.81. WHR, Plat, and MCHC correlated moderately (*r* < 0.7). Thus, we conducted a genetic association study of the ABO gene region with the 5 unrelated hematological traits.

**Table 1:** Summary of participant characteristics and hematological traits

**Variables**	**Mean ± standard deviation**
Sample size (n)	6675
Age (years)	50.1 ± 8.8
Male (%)	47.7
Ansung (%)	50.5
WBC count (10^3^/μl)	6.4 ± 1.9
RBC count (10^6^/μl)	4.4 ± 0.4
Hb (g/dl)	13.8 ± 1.6
Hct (%)	41.2 ± 4.4
Platelet count (10^3^/μl)	244.3 ± 60.1
MCV (fl)	93.9 ± 5.3
MCH (pg)	31.2 ± 2.0
MCHC (g/dl)	33.2 ± 1.3

*Note. WBC*: white blood cell, *RBC*: red blood cell, *Hb*: hemoglobin, *Hct*: hematocrit, *MCV*: mean corpuscular volume, *MCH*: mean corpuscular hemoglobin, *MCHC*: mean corpuscular hemoglobin concentration.

### SNP selection

SNPs in Affymetrix 5.0 SNP array and imputation SNP data were obtained from the Korean Genome Epidemiology Study (KoGES) of the National Institute of Health, Korea, and the genotype data were Korea Association Resource consortium (KARE) data. The genomewide SNPs have been examined in genomewide association studies for anthropometric [11] and biochemical traits [12]. In this study, we focused on the *ABO* region that was reported by a Japanese study. Population stratification of the genotyped samples was also tested in an earlier report [11]; there was no population stratification that was demonstrated by Multidimensional Scaling (MDS) Analysis and Principal Component Analysis (PCA) (Additional file 1: Figure S1). Genomic inflation factors were low ranging from 1.01 (WBC) to 1.03 (Hct), suggesting that population stratification was well controlled (Additional file 2: Table S1)

We initially used 76 SNPs around *ABO* on chromosome 9 from 135,070 kbp to 135,152 kbp. The ABO gene boundaries were established by linkage disequilibrium (LD) analysis (Additional file 3: Figure S2). Three LD blocks encompassed *ABO* and its promoter region. The 3 LD blocks included 58 SNPs, 10 of which were genotyped by Affymetrix 5.0 SNP array; the remaining 48 SNPs were imputed by IMPUTE, based on the HAPMAP database. The characteristics of the 58 SNPs are described in Additional file 2: Table S1. The SNPs were classified as 8 nonsynonymous SNPs, 1 synonymous SNP, 8 upstream SNPs, and 41 intron SNPs.

### ABO gene SNP association study

For the association analysis, we isolated 6 tagging SNPs for *ABO*. In Additional file 4: Table S2, we describe the 6 SNP groups with high LD (r^2^ > 0.9) and underlined the tagging SNPs. The association results are described in Table 2. In this study, we used Bonferroni correction *p*-value criteria (< 8.3 × 10^-3^) for multiple comparisons, and the significant effect sizes and p-values are underlined in Table 2. Three SNPs (rs2073823, rs8176720, and rs495828) and 3 SNPs (rs2073823, rs8176717, and rs687289) were significantly associated with RBC and MCV, respectively.

**Table 2:** Association analysis of 6 high-LD-group tagging SNPs with five hematological traits by linear regression analysis, controlling for area, age, and sex as covariates

				**WBC**		**RBC**		**Plat**		**MCV**		**MCHC**	
**CHR**	**SNP**	**BP**	**A1**	**BETA ± SE**	***P***	**BETA ± SE**	***P***	**BETA ± SE**	***P***	**BETA ± SE**	***P***	**BETA ± SE**	***P***
9	rs2073823	135122337	A	0.056 ± 0.039	0.15	0.036 ± 0.008	2.13 × 10^-6†^	−1.213 ± 1.253	0.33	−0.480 ± 0.118	5.06 × 10^-5†^	0.075 ± 0.029	9.55 × 10^-3^
9	rs8176720	135122694	C	0.043 ± 0.032	0.19	0.018 ± 0.006	5.58 × 10^-3^	−0.232 ± 1.033	0.82	−0.071 ± 0.098	0.47	0.025 ± 0.024	0.30
9	rs8176717	135122855	T	0.001 ± 0.039	0.97	−0.012 ± 0.008	0.13	0.885 ± 1.253	0.48	0.356 ± 0.118	2.62 × 10^-3†^	−0.035 ± 0.029	0.22
9	rs687289	135126927	A	0.001 ± 0.032	0.97	0.001 ± 0.006	0.89	1.017 ± 1.025	0.32	−0.274 ± 0.097	4.59 × 10^-3^	0.020 ± 0.024	0.40
9	rs8176681	135129575	C	0.016 ± 0.035	0.64	0.009 ± 0.007	0.19	−1.488 ± 1.109	0.18	0.063 ± 0.105	0.55	0.003 ± 0.026	0.92
9	rs495828	135144688	T	−0.050 ± 0.037	0.18	−0.030 ± 0.007	2.69 × 10^-5†^	2.217 ± 1.180	0.06	0.061 ± 0.112	0.58	−0.042 ± 0.027	0.12

*Note. Chr*: chromosome, *BP*: base pair, *A1*: minor allele, *WBC*: white blood cell count, *RBC*: red blood cell count, Platelet, *MCV*: mean corpuscular volume, *MCHC*: mean corpuscular hemoglobin concentration.

Underlined p-values were passed the multiple correction criteria of Bonferroni correction (p<0.008).

^†^Significant result after adjusted by rs2073823.

To identify independent association signals, we performed a conditional analysis by including rs2073823 in the linear regression model of other significant SNP associations. For RBC, the association signal of rs8176720 disappeared (*p*-value = 0.803), but that of rs495828 was significant (*p*-value = 0.004) after adjusting for rs2073823. rs8176717 was moderately associated with MCV (*p*-value = 0.045), but the association signal with rs687289 disappeared (*p*-value =0.492). Thus, we identified 3 independent associations (rs2073823, rs8176717 and rs495828) between ABO and hematological traits.

### Identification of copy number variation

**Figure 1:**
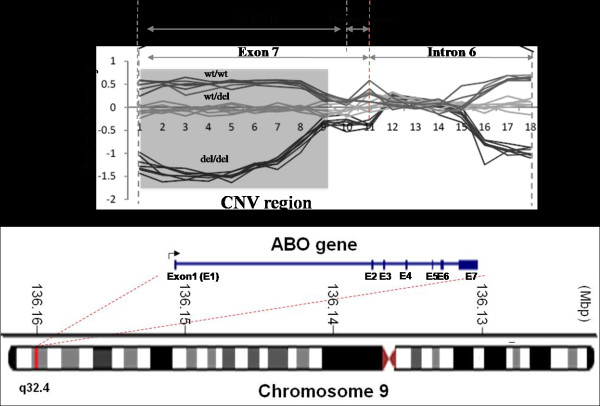
**Probe intensity of copy number variation region: Log**^**2**^**ratio plot of the test sample and the reference (NA10851) signal intensity.**

A copy number variation (CNV) region was detected on chromosome 9, 135,120,477–135,122,527 (Figure 1), which includes the 3′ untranslated region of the ABO gene. Because the array CGH experiment was conducted using a subset (n = 4694) of all KoGES samples, to maximize the sample size, we surveyed a tagging SNP that correlated well with CNV region genotypes. We determined the SNP rs8176717 to correlate with the CNV region (*r*^2^ = 0.96), the minor allele of which (T allele) implied the minor allele (deletion allele) of CNV.

#### **Haplotype analysis**

We estimated the haplotyes for the 6 SNPs (Table 3). A total of 6 haplotypes were predicted, comprising 4 common haplotypes and 2 rare haplotypes (frequencies < 0.05). A haplotype (Hap 3) included the minor allele of rs8176717, which tagged the CNV and was significantly associated with MCV (beta ± se = 0.363 ± 0.118, *p*-value = 2.09 × 10^-3^). The other significant haplotype was Hap 4, which was linked to RBC (beta ± se = 0.036 ± 0.008, p-value = 4.27 × 10^-6^) and MCV (beta ± se = −0.512 ± 0.119, p-value = 1.81 × 10^-5^).

**Table 3:** Haplotype frequencies and association results of six SNPs with red blood cell count (RBC) and mean corpuscular volume (MCV)

**Haplotypes**	**Tagging SNPs for ABO gene region**						**Haplotype frequency**	**RBC**		**MCV**	
	**rs2073823**	**rs8176720**	**rs8176717**	**rs687289**	**rs8176681**	**rs495828**		**beta ± se**	***p***	**beta ± se**	***p***
Hap 1	G	T	G	G	C	G	0.297	0.008 ± 0.007	0.249	0.064 ± 0.106	0.544
Hap 2	G	T	G	A	T	T	0.261	−0.030 ± 0.007	2.63 × 10^-5^	0.064 ± 0.111	0.563
Hap 3	G	C	T	G	T	G	0.214	−0.013 ± 0.008	0.103	0.363 ± 0.118	2.09 × 10^-3^
Hap 4	A	C	G	A	T	G	0.208	0.036 ± 0.008	4.27 × 10^-6^	−0.512 ± 0.119	1.81 × 10^-5^
Hap 5	G	T	G	G	T	G	0.012	NA	NA	NA	NA
Hap 6	A	C	G	A	C	G	0.007	NA	NA	NA	NA

## Discussion

In this study, we confirmed the association between *ABO* and hematological traits in a large Korean population. Also, we found a copy number variation that influenced hematological traits.

Of the 6 tagging SNPs in the ABO gene, rs2073823 was the most significant, in perfect LD (r^2^ = 0.995) with rs8176746, an SNP from the Japanese GWAS on hematological traits [8]. The minor allele of rs8176746 is the variant that encodes the B-type blood group. [10]. However, this SNP was not reported in a GWAS of hematological traits in Caucasians [7,9], possibly due to ethnic differences in the minor allele frequency in Caucasian (0.08), Chinese (0.23), and Japanese (0.17) individuals. The allele frequencies correspond well to the frequency of blood type B in Caucasian (~8%) and East Asian (~22%) individuals, as inferred from the BLOODBOOK website (http://www.bloodbook.com/world-abo.html). Using the minor allele frequency (0.008) and the mean RBC (± sd) = 4.82 ± 0.50 of Caucasians, we estimated the number of individuals required for the 80% power at the alpha = 5 × 10^-8^ (genome-wide significant levels) [7,9]. To be replicated the rs2073823 (LD with rs8176746) association, 51,876 individuals would be necessary. However, the previous European study [7] used 33,623 individuals which it was smaller than the estimated individual number at the genome-wide significant level. In our study, individuals with a minor allele of rs2073823 had elevated RBC counts but decreased MCV. Thus, individuals with the blood type B might have higher RBC counts and lower MCV than those with other blood types, at least among Asians.

The second highest signal was generated from an upstream SNP, rs495828, which was also was reported in the Japanese GWAS [8]; this SNP was in perfect LD with rs651007, which was reported in an African-American GWAS [9]. Notably, the 3 proximal SNPs (rs651007, rs579459, and rs649129) were in complete LD (r^2^ = 0.99) with rs495828. Because carriers of the minor allele of these 3 SNPs have significantly lower levels of sP-selectin [5], sE-selectin [6], and risk of CAD [4], the relationship between hematological traits and coronary artery disease phenotypes should be examined.

The Japanese GWAS reported complete LD between rs8176746 and rs495828. To confirm the LD, we estimated the LD in Europeans (r^2^ = 0.010 and D’ = 0.150), Africans (r^2^ = 0.035 and D’ = 1.000), Chinese, Japanese (r^2^ = 0.050 and D’ = 1.000), and Koreans in this study (r^2^ = 0.087 and D’ = 1.000). Even though it was reported that rs8176746 and rs495828 are in complete LD in the Japanese study, the data from publically available databases suggests some inconsistencies with high D’ and low r^2^. This suggests that rs495828 may represent an independent association signal for RBC. A limitation of our study is that the 2 most significant SNPs—rs8176746 and rs495828—were not genotyped directly, although the minor allele frequencies of these SNPs are similar to those reported in the Japanese GWAS [2].

The CNV region that we identified has been reported by 7 other studies [13-18]. The minor allele of CNV was a deletion mutation of the 3′ untranslated region of ABO; thus, the CNV might influence its expression. In our results, the haplotype included the minor allele of the CNV-tagging SNP (rs8176717) and was significant associated with MCV. This result is notable, because most GWASs do not evaluate the link between a CNV and phenotype traits. Thus, our study is a model that can be used to correlate SNPs and CNV.

## Conclusions

ABO is one of the genetic factors that are associated with hematological traits in East Asian populations. Also, we identified a novel association with a SNP that tags a common CNV with MCV. This result is notable, because GWASs fail to evaluate the link between a CNV and phenotype traits.

## Methods

### Study participants

This study was conducted as part of an ongoing population-based cohort of the Korean Genome and Epidemiology Study (KoGES). All participants were recruited from the cities of Ansung and Ansan in Gyeonggi-do Province, Korea. This study was approved by the Institutional Review Board of the Korea National Institute of Health, and all participants provided written informed consent for study participation.

### Hematological trait measures

A total of 6675 samples were available for hematological trait analysis, as described in Table 1, Venous blood samples were drawn from all participants into 4.5-ml tubes that contained K3-EDTA as an anticoagulant and were analyzed within 30 min to 4 h of collection. Hematological traits were measured by Seoul Clinical Laboratories Company Ltd. The ADIVA 120 hematology system (Bayer Diagnostics, USA) was calibrated per the manufacturer’s guidelines. WBC count, RBC count, platelet count, Hb level, Hct, mean corpuscular volume (MCV), mean corpuscular hemoglobin (MCH) level, and mean corpuscular hemoglobin concentration (MCHC) were determined automatically for all samples.

### SNP determination

The ABO gene is located on chromosome 9 from 135,120,384–135,140,451 bp. SNP genotypes were determined using the Affymetrix 5.0 SNP array, the experimental procedures of which are detailed elsewhere [11]. Further, to increase the number of genotype markers, we imputed additional SNPs using the Affymetrix 5.0 SNP array and the HapMap database (HAPMAP 3, http://www.hapmap.org); the imputation methods have been described [19]. The final SNPs were selected using the following criteria: minor allele frequency > 0.1; missing rate < 10%; and Hardy-Weinberg equilibrium test *p*-value > 0.05 for experimentally determined SNPs and imputation SNPs. Information on the SNPs was obtained from the dbSNP database (http://www.ncbi.nlm.gov/snp), and the genetic distance between the Korean and other populations was calculated using F-statistic [20]. LD blocks and pairwise LD (D’ and r^2^) of SNPs were estimated and determined for the tagging SNPs in the ABO gene region using Haploview [21].

### CNV determination

To identify regions of CNV, samples from 4694 participants were genotyped using the NimbleGen HD2 2x720K array comparative genomic hybridization (aCGH) assay with DNA from peripheral blood. All samples passed experimental quality control metrics, such as the chromosome X shift and mad.1dr, as determined using NimbleScan version 2.5 per the manufacturer’s guidelines. After quality control procedures, the signal intensity ratio between the test and reference sample (NA10851 from the HapMap cell line DNA) of each probe was log2-transformed.

Regions of CNV were identified using the Genome Alteration Detection Analysis algorithm [22], which was used for samples from 4694 participants, with T = 10, alpha = 0.2, and MinSegLen = 10. The threshold for defining regions of CNV was set to an average log2 ratio of ± 0.25 Additional file 5: Figure S3.

### CNV-tagging SNP

We tagged SNPs to maximize the sample size. To find SNPs that tagged the identified CNVs well, we performed a correlation analysis that was similar to that in the Wellcome Trust Case Control Consortium CNV study [23] using calls that were identified in a GWAS with the Affymetrix 5.0 array [11]. For each CNV, we calculated the squared Pearson's r value between CNV regions and SNPs. We considered all SNPs within 1 Mb of the estimated 2 breakpoints (i.e., start and end points) of each CNV region. We selected the SNP with the highest r^2^ value for each CNV region.

### Association tests

Linear regression analysis was used to analyze the association between ABO SNPs or haplotypes of tagging SNPs and hematological trait, controlling for gender, age, and recruitment center as covariates. The asymptotic Hardy-Weinberg equilibrium test was conducted using PLINK (version 1.07) [24], and all reported p-values were two-sided (α = 0.05). Associations between SNPs and hematological traits were significant at *p* < = 8.3 × 10^-3^ after Bonferroni correction for multiple testing of 6 SNPs. The sample size was estimated for rs2073823 association in the European with the 80% statistical power at the genome-wide significance level by the QUANTO software (version 1.2.4, http://hydra.usc.edu/gxe/).

## Authors’ contributions

KWH participated in the design of the study, genetic analysis, and drafted the manuscript. SM participated in the design of the study, CNV genotype determination, and drafted the manuscript. YJK participated in the CNV-tagging SNP identification and statistical analysis. YKK participated in the CNV genotype experiments. DJK participated in the SNP and CNV genotype experiments. CK participated in the hematological trait measurements. SSK participated in writing the manuscript and discussion. BJK participated in writing the manuscript and discussion.

## Acknowledgments

This work was supported by an intramural grant from the Korea National Institute of Health (2010-N73001-00) and by grants from the Korea Centers for Disease Control and Prevention (4845–301, 4851–302, and 4851–307).

## Supplementary Material

Additional file 1 **Figure S1.** Multidimensional scaling (MDS) analysis and principal component analysis (PCA) [Cho et al., 2009]. Click here for file

Additional file 2 **Table S1.** Genomic inflation factor for hematological trait genome-wideassociation studies. Click here for file

Additional file 3 **Figure S2.** Linkage disequilibrium blocks of ABO gene region. Click here for file

Additional file 4 **Table S2.** SNP list of ABO gene region, minor allele frequency comparison, and genetic distance calculation between KARE and other populations. Underlined SNPs indicate the tagging SNPs for the ABO gene region used in the main paper. Click here for file

Additional file 5 **Figure S3. CNV clustering results.** We used CNV tools to summarize the signal intensity data and assign a [specific OR discrete] CNV genotype within the CNV region. (A) Histogram of the clustering procedure using data, transformed by the linear discriminant function (LDF). (B) Cluster plot of the CNV region predicted from the LDF signal. Click here for file

## References

[B1] Storry JR Olsson ML The ABO group system revisited: review and update Immunohematology 2009 25 48 59 19927620

[B2] Yamamoto F McNeill PD Hakomori S Genomic organization of human histo-blood group ABO genes Glycobiology 1995 5 51 58 10.1093/glycob/5.1.51 7772867

[B3] Schunkert H König IR Kathiresan S Reilly MP Assimes TL Holm H Preuss M Stewart AF Barbalic M Gieger C Large-scale association analysis identifies 13 new susceptibility loci for coronary artery disease Nat Genet 2011 43 333 338 10.1038/ng.784 21378990 PMC3119261

[B4] Qi L Cornelis MC Kraft P Jensen M van Dam RM Sun Q Girman CJ Laurie CC Mirel DB Hunter DJ Genetic variants in ABO blood group region, plasma soluble E-selectin levels and risk of type 2 diabetes Hum Mol Genet 2010 19 1856 1862 10.1093/hmg/ddq057 20147318 PMC2850622

[B5] Paterson AD Lopes-Virella MF Waggott D Boright AP Hosseini SM Carter RE Shen E Mirea L Bharaj B Sun L Genome-wide association identifies the ABO blood group as a major locus associated with serum levels of soluble E-selectin Arterioscler Thromb Vasc Biol 2009 29 1958 1967 10.1161/ATVBAHA.109.192971 19729612 PMC3147250

[B6] Barbalic M Dupuis J Dehghan A Bis JC Hoogeveen RC Schnabel RB Nambi V Bretler M Smith NL Peters A Large-scale genomic studies reveal central role of ABO in sP-selectin and sICAM-1 levels Hum Mol Genet 2010 19 1863 1872 10.1093/hmg/ddq061 20167578 PMC2850624

[B7] Ganesh SK Zakai NA van Rooij FJ Soranzo N Smith AV Nalls MA Chen MH Kottgen A Glazer NL Dehghan A Multiple loci influence erythrocyte phenotypes in the CHARGE Consortium Nat Genet 2009 41 1191 1198 10.1038/ng.466 19862010 PMC2778265

[B8] Kamatani Y Matsuda K Okada Y Kubo M Hosono N Daigo Y Nakamura Y Kamatani N Genome-wide association study of hematological and biochemical traits in a Japanese Population Nat Genet 2010 42 210 215 10.1038/ng.531 20139978

[B9] Lo KS Wilson JG Lange LA Folsom AR Galarneau G Ganesh SK Grant SF Keating BJ McCarroll SA Mohler ER 3rd Genetic association analysis highlights new loci that modulate hematological trait variation in Caucasians and African Americans Hum Genet 2011 129 307 317 10.1007/s00439-010-0925-1 21153663 PMC3442357

[B10] Yip SP Sequence variation at the human ABO locus Ann Hum Genet 2002 66 1 27 10.1017/S0003480001008995 12014997

[B11] Cho YS Go MJ Kim YJ Heo JY Oh JH Ban HJ Yoon D Lee MH Kim DJ Park M A large-scale genome-wide association study of Asian population uncover genetic factors influencing eight quantitative traits Nat Genet 2009 41 527 534 10.1038/ng.357 19396169

[B12] Kim YJ Go MJ Hu C Hong CB Kim YK Lee JY Hwang JY Oh JH Kim DJ Kim S Large-scale genome-wide association studies in East Asians identify new genetic loci influencing metabolic traits Nat Genet 2011 43 990 995 10.1038/ng.939 21909109

[B13] Itsara A Cooper GM Baker C Girirajan S Li J Absher D Krauss RM Myers RM Ridker PM Chasman DI Population analysis of large copy number variants and hotspots of human genetic disease Am J Hum Genet 2009 84 148 161 10.1016/j.ajhg.2008.12.014 19166990 PMC2668011

[B14] Jakobsson M Scholz SW Scheet P Gibbs JR VanLiere JM Fung HC Szpiech ZA Degnan JH Wang K Guerreiro R Genotype, haplotype and copy-number variation in worldwide human populations Nature 2008 451 998 1003 10.1038/nature06742 18288195

[B15] Conrad DF Pinto D Redon R Feuk L Gokcumen O Zhang Y Aerts J Andrews TD Barnes C Campbell P Origins and functional impact of copy number variation in the human genome Nature 2010 464 704 712 10.1038/nature08516 19812545 PMC3330748

[B16] McCarroll SA Kuruvilla FG Korn JM Cawley S Nemesh J Wysoker A Shapero MH de Bakker PI Maller JB Kirby A Integrated detection and population-genetic analysis of SNPs and copy number variation Nat Genet 2008 40 1166 1174 10.1038/ng.238 18776908

[B17] Shaikh TH Gai X Perin JC Glessner JT Xie H Murphy K O’Hara R Casalunovo T Conlin LK D’Arcy M High-resolution mapping and analysis of copy number variations in the human genome: a data resource for clinical and research applications Genome Res 2009 19 1682 1690 10.1101/gr.083501.108 19592680 PMC2752118

[B18] Wang K Li M Hadley D Liu R Glessner J Grant SF Hakonarson H Bucan M PennCNV: an integrated hidden Markov model designed for high resolution copy number variation detection in whole-genome SNP genotyping data Genome Res 2007 17 1665 1674 10.1101/gr.6861907 17921354 PMC2045149

[B19] Hong KW Lim JE Kim YJ Cho NH Shin C Oh B KARE Genomewide Association Study of Blood Pressure Using Imputed SNPs Genomics & Informatics 2010 8 103 107 10.5808/GI.2010.8.3.103

[B20] Wright S The genetical structure of populations Nature 1950 15 323 354 10.1111/j.1469-1809.1949.tb02451.x 24540312

[B21] Barrett JC Fry B Maller J Daly MJ Haploview: analysis and visualization of LD and haplotype maps Bioinformatics 2005 28 1323 1328 10.1093/bioinformatics/bth457 15297300

[B22] Pique-Regi R Monso-Varona J Ortega A Seeger RC Triche TJ Asgharzadeh S Sparse representation and Bayesian detection of genome copy number alterations from microarray data Bioinformatics 2008 24 309 318 10.1093/bioinformatics/btm601 18203770 PMC2704547

[B23] Craddock N Hurles ME Cardin N Pearson RD Plagnol V Robson S Vukcevic D Barnes C Conrad DF Wellcome Trust Case Control Consortium Genome-wide association study of CNVs in 16,000 cases of eight common diseases and 3,000 shared controls Nature 2010 464 713 720 10.1038/nature08979 20360734 PMC2892339

[B24] Purcell S Neale B Todd-Brown K Tomas L Ferreira MA Bender D Maller J Sklar P de Bakker PI Daly MJ PLINK: a tool set for whole-genome association and population based linkage analyses Am J Hum Genet 2007 81 559 575 10.1086/519795 17701901 PMC1950838

