# Supplementary figures and images for: Association between the ABO locus and hematological traits in Korean
Source: BMC Genet. 2012 Sep 10;13:78. doi: 10.1186/1471-2156-13-78 (PMC3472170; doi:10.1186/1471-2156-13-78)

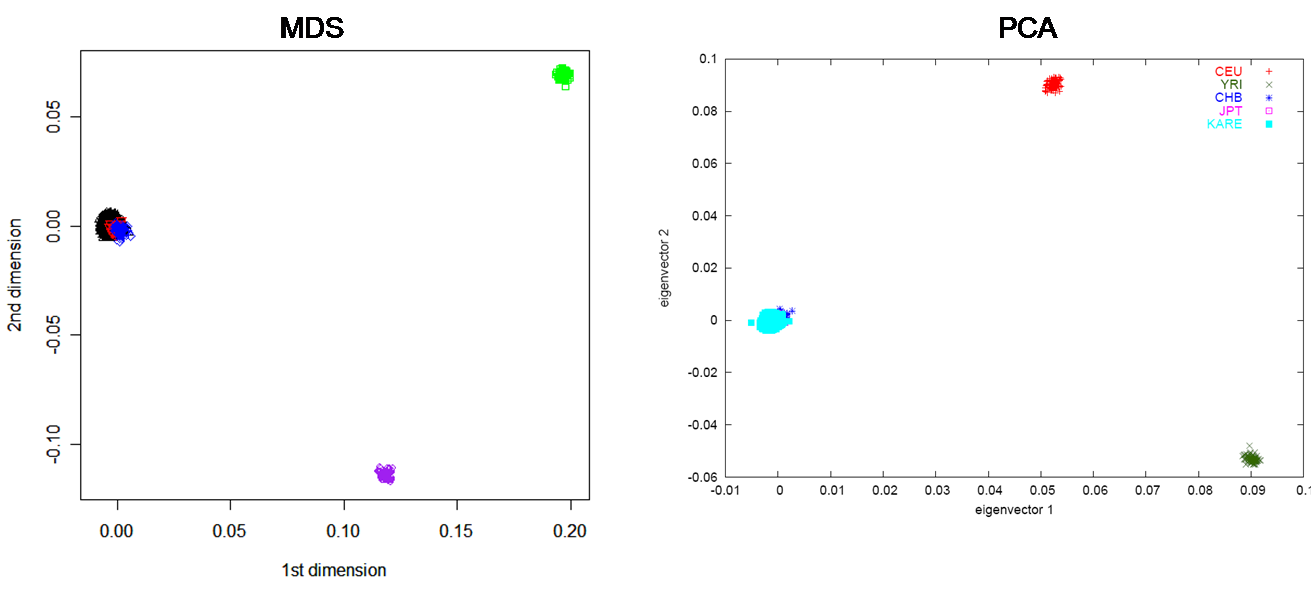

Supplement: Additional file 1 — Figure S1. Multidimensional scaling (MDS) analysis and principal component analysis (PCA) [Cho et al., 2009]. [file 1471-2156-13-78-S1.tiff]

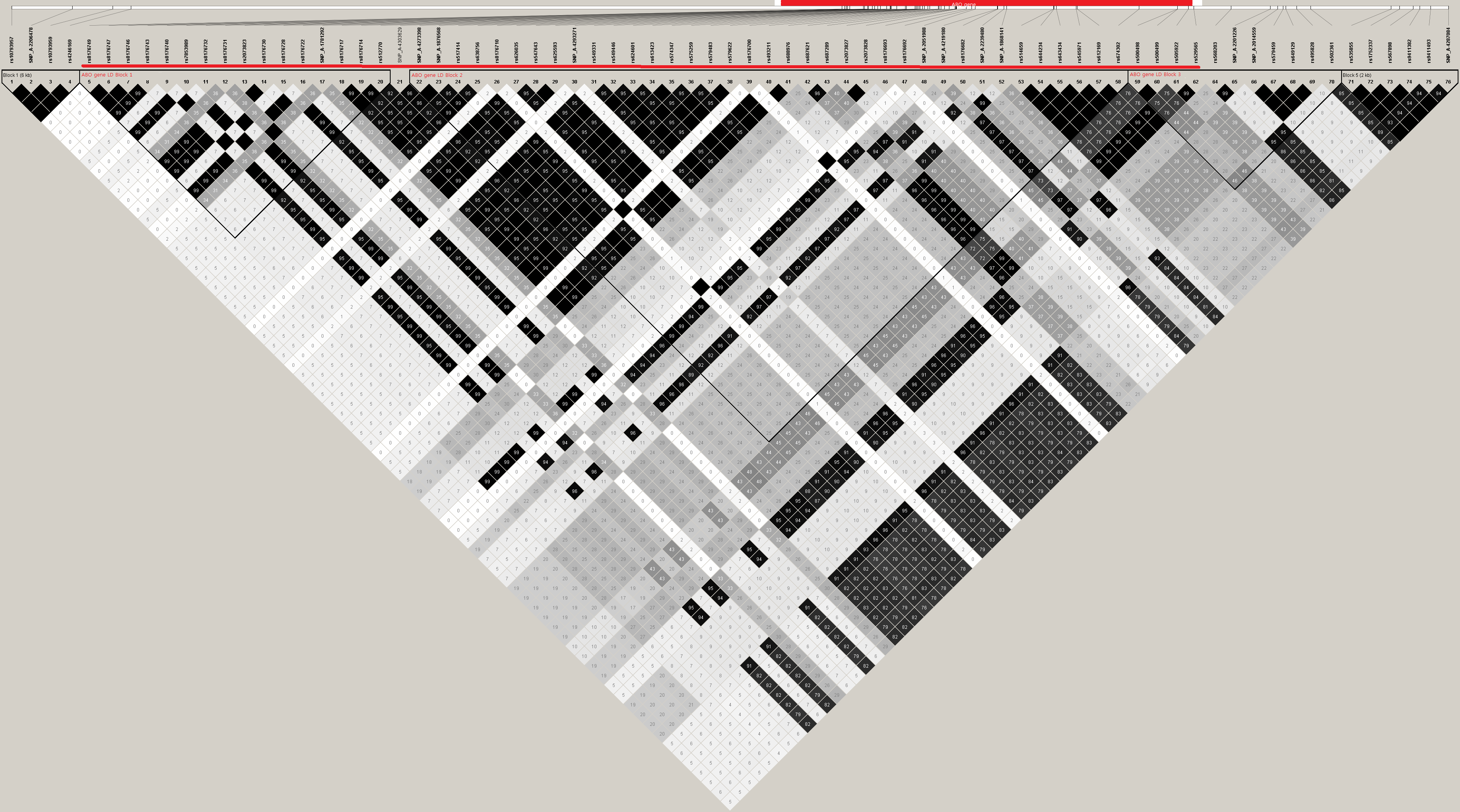

Supplement: Additional file 3 — Figure S2. Linkage disequilibrium blocks of ABO gene region. [file 1471-2156-13-78-S3.tiff]

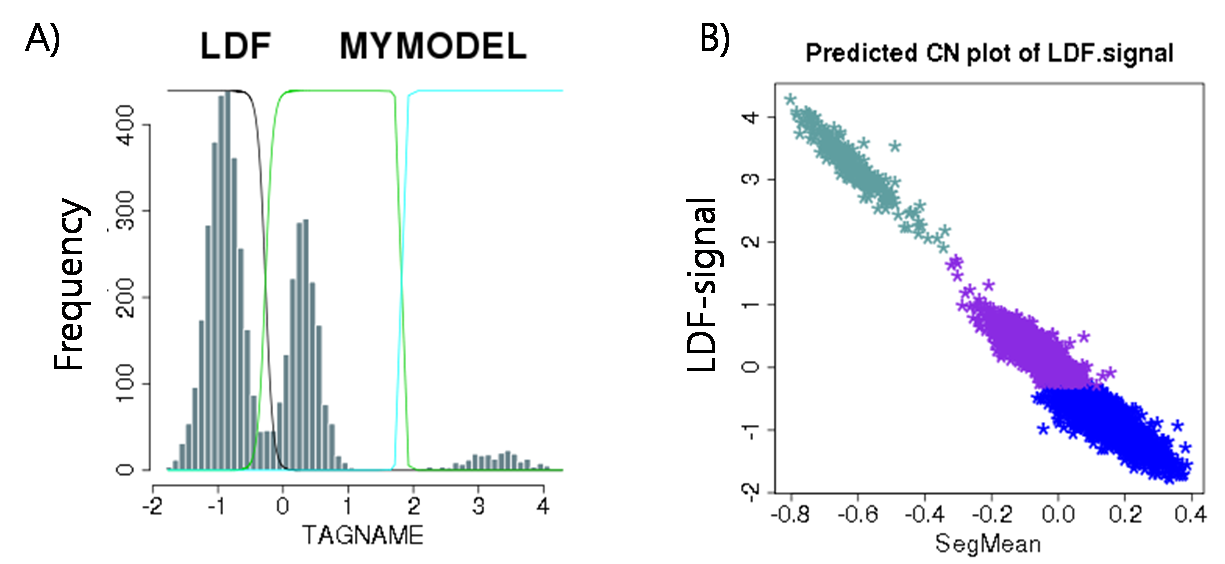

Supplement: Additional file 5 — Figure S3. CNV clustering results. We used CNV tools to summarize the signal intensity data and assign a [specific OR discrete] CNV genotype within the CNV region. (A) Histogram of the clustering procedure using data, transformed by the linear discriminant function (LDF). (B) Cluster plot of the CNV region predicted from the LDF signal. [file 1471-2156-13-78-S5.tiff]
